# Oestradiol levels and superoxide dismutase activity in age-related cataract: a case–control study

**DOI:** 10.1186/s12886-016-0392-0

**Published:** 2016-11-29

**Authors:** Dragana Škiljić, Staffan Nilsson, Anne Petersen, Jan-Olof Karlsson, Anders Behndig, Lada Kalaboukhova, Madeleine Zetterberg

**Affiliations:** 1 Department of Clinical Neuroscience/Ophthalmology, Institute of Neuroscience and Physiology, The Sahlgrenska Academy at University of Gothenburg, Gothenburg, Sweden; 2 Department of Ophthalmology, Sahlgrenska University Hospital, Mölndal, Sweden; 3 Department of Mathematical Statistics, Institute of Mathematical Sciences, Chalmers University of Technology, Gothenburg, Sweden; 4 Department of Medical Chemistry and Cell Biology, Institute of Biomedicine, The Sahlgrenska Academy at University of Gothenburg, Gothenburg, Sweden; 5 Department of Clinical Sciences/Ophthalmology, Umeå University, Umeå, Sweden

**Keywords:** Age-related cataract, Gender, Oestradiol, Superoxide dismutase, Oxidative stress

## Abstract

**Background:**

It has been suggested that the higher prevalence of cataract in women is caused by a withdrawal effect of oestrogen at menopause. In vitro studies have demonstrated protection of serum oestradiol (E2) against oxidative stress through upregulation of antioxidant enzymes, including superoxide dismutase (SOD). The purpose of the present study was to investigate E2 levels and SOD erythrocyte activity in patients with age-related cataract.

**Methods:**

The studied subjects consisted of 103 patients with age-related cataract and 22 controls. Cataracts were classified as nuclear, cortical, or posterior subcapsular. Blood samples were collected and data on smoking, hormonal use, diabetes and age at menarche/menopause was obtained for all individuals. Serum oestradiol analyses were performed with radioimmunoassay (RIA) and SOD activity was measured in erythrocyte lysates.

**Results:**

A negative correlation between age and E2 concentration was seen in a linear regression analysis. No correlation was seen between SOD activity and age or gender and no correlation between E2 levels and SOD activity was found using multiple linear regression. The mean level of E2 for all male subjects was 50.1 ± 16.3 pmol/L, significantly higher compared to 13.8 ± 11.8 pmol/L for postmenopausal women.

**Conclusion:**

The present study does not support a role for E2-induced effects on SOD in cataract formation. The findings of higher E2 levels in men than in postmenopausal women may suggest that decreased oestrogen at menopause is partially responsible for the gender-related difference in cataract prevalence. However, the latter can only be verified through prospective randomized trials using hormonal replacement therapy.

## Background

Cataract is the most common cause of blindness in the world and as lifespan is getting longer, an increased number of people requiring cataract surgery can be anticipated. Several studies report higher prevalence of lens opacities in women as compared to men of the same age [1–3]. It has been proposed that with the dramatic reduction in oestradiol levels in women entering menopause, the potentially protective effects of oestrogen are withdrawn, resulting in increased risk of cataract in women. Studies have shown that earlier menarche and later menopause, causing an extended period of reproductive years, are associated with lower risk of cataract and that the use of exogenous oestrogens, *i.e.* hormonal replacement therapy (HRT), is associated with decreased risk of cataract. It has therefore been suggested that oestrogen may protect against cataract. The Blue Mountain Eye study reported that current HRT users had lower risk of developing cortical cataract and this was also confirmed in the follow-up study [4]. Current and longer use of HRT showed lower incidence of nuclear lens opacities in both the Beaver Dam Eye Study and the Framingham Eye Study cohort [5, 6].

Oxidative stress is a key factor in cataract formation. The major oestrogen, oestradiol (E2), has been ascribed antioxidant properties and several studies have shown oestrogen-mediated protection against oxidative stress through upregulation of antioxidant enzymes including superoxide dismutase (SOD) [7–9]. In addition, lens epithelial cells have also shown increased SOD activity after exposure to E2 [10]. Therefore, the purpose of the present study was to investigate possible correlations between SOD activity and E2 levels in patients with age-related cataract and controls.

## Methods

### Patients

Patients with age-related cataract and controls were recruited from the Eye Clinic at Sahlgrenska University Hospital/Mölndal, after informed consent. The Regional Research Ethics Committee in Gothenburg approved the study and the tenets of the Declaration of Helsinki were followed. The studied subjects consisted of 103 patients with age-related cataract and 22 controls. Cataracts were classified as nuclear, cortical, or posterior subcapsular using biomicroscopy performed by four different surgeons. Patients with secondary cataracts, such as steroid-induced cataract, were excluded. Controls were either spouses or relatives to glaucoma patients, had glaucoma themselves or were recruited from the emergency ophthalmic clinic, with diagnoses such as vitreous detachment or blepharitis. Exclusion criteria for controls were previous cataract surgery, age <60 years or current lens opacities. The latter was evaluated by slit lamp biomicroscopy by one ophthalmologist only. Data on smoking (current/former smoking habits), hormones (current/former use of contraceptives and/or hormone replacement therapy), diabetes and age at menarche/menopause was obtained for all individuals and age was reported at the time of sample collection (Table 1).

**Table 1:** Demographics of studied patients with age-related cataract and controls

Parameter	Cataract	Controls	*p*-value
Age (years)	*n = 103*	*n = 22*	
	71 ± 10.9	70 ± 3.4	0.35
Sex	*n = 103*	*n = 22*	
Female	61 (59.2)	16 (72.7)	0.24
Male	42 (40.8)	6 (27.3)	
Type of lens opacity^a^	*n = 100*		
Nuclear	93 (93.0)	-	-
Cortical	42 (42.0)	-	
Posterior subcapsular	23 (23.0)	-	
Mixed cataract	*n = 102*		
Yes	51 (50.0)	-	-
No	51 (50.0)	-	
Diabetes	*n = 103*	*n = 22*	
Yes	20 (19.4)	-	0.02
No	83 (80.6)	22 (100)	
Smoking	*n = 103*	*n = 22*	
Current smoker	19 (18.4)	1 (4.5)	0.11
Ever smoker^b^	69 (67.0)	12 (54.5)	0.27
Hormonal status	*n = 102*	*n = 18*	
Currently on hormones	9 (8.8)	3 (16.7)	0.31
Ever on hormones^b^	43 (42.2)	11 (61.1)	0.14
Menarche / Menopause			
Age at menarche	*n = 57*	*n = 12*	
	13 ± 1.3	13 ± 1.4	0.64
Age at menopause	*n = 50*	*n = 13*	
	49 ± 6.8	50 ± 3.6	0.49
Years in menopause^c^	*n = 50*	*n = 13*	
	24 ± 11.7	20 ± 5.8	0.16
Reproductive years	*n = 48*	*n = 12*	
	35 ± 6.9	38 ± 3.2	0.27

Data presented as absolute numbers (%) or mean ± SD. *P*-values were calculated with Pearson’s chi-square test for categorical parameters and Student’s *t*-test for continuous parameters

^a^ Both eyes included. Many individuals had more than one subtype, *i.e.* mixed cataract

^b^ Includes former and current users

^c^ Years in menopause prior to sampling

### Blood sampling

Venous blood samples were drawn from patients and controls and collected using tubes with anticoagulant (EDTA) for whole blood samples and tubes with clot activator and gel separator for serum samples (VACUETTE, Greiner Bio-One, Kremsmünster, Austria). The whole blood samples were handled the same day and erythrocytes were collected after centrifugation at 800 × g for 10 min at 4 °C. The erythrocytes were then lysed in four times its volume of ice-cold distilled water after which the samples were centrifuged at 10.000 × g for 15 min at 4 °C and erythrocyte lysate was collected. The serum sample tubes were centrifuged at 1800 × g for 10 min after coagulation, within 3 h after sampling. Both aliquots of serum and erythrocyte lysate were stored at −80 °C until assayed.

### Oestradiol levels

Serum oestradiol analyses were performed with radioimmunoassay (Spectria Estradiol RIA, Orion Diagnostica, Espoo, Finland) with an extraction step prior to quantification, yielding a sensitivity of 4 pmol/L, previously described by Ankarberg-Lindberg et al. [11]. The assay measures total oestradiol (17β-oestradiol) serum concentrations *i.e.* both free E2 and E2 previously bound to sex hormone-binding globulin (SHBG) or albumin.

### Total superoxide dismutase activity

SOD activity was measured in erythrocyte lysate samples using the Superoxide Dismutase Assay kit, according to the manufacturer’s protocol (Cayman Chemical Company, Ann Arbor, MI, USA). Absorbance was measured at 440 nm on the microplate reader Infinite M200 PRO (Tecan group Ltd., Männedorf, Switzerland) using 96-well microplates (Greiner Bio-One, Kremsmünster, Austria). The SOD assay uses tetrazolium salt to detect superoxide radicals generated by xanthine oxidase and hypoxanthine. One unit (U) of SOD is defined as the amount of enzyme needed to execute 50% dismutation of the superoxide radical. The assay measures total SOD activity per millilitre erythrocyte lysate (U/ml).

### Statistical analysis

Demographic differences between cataract patients and control subjects were analysed using Student’s *t*-test for continuous parameters and Pearson’s chi-square test for categorical parameters (Table 1). Multiple linear regression was used to analyse potential predictors of the dependent variables; E2 levels and SOD activity. In the initial multivariate analysis, cataract type, smoking, diabetes, age at menarche/menopause and hormonal status were included, but since they showed no statistical significance they were excluded in the final model. Statistical analyses were performed using IBM SPSS Statistics 21.0 (IBM Corp., Armonk, NY, USA) and *p*-values ≤0.05 were considered statistically significant.

## Results

**Fig. 1:**
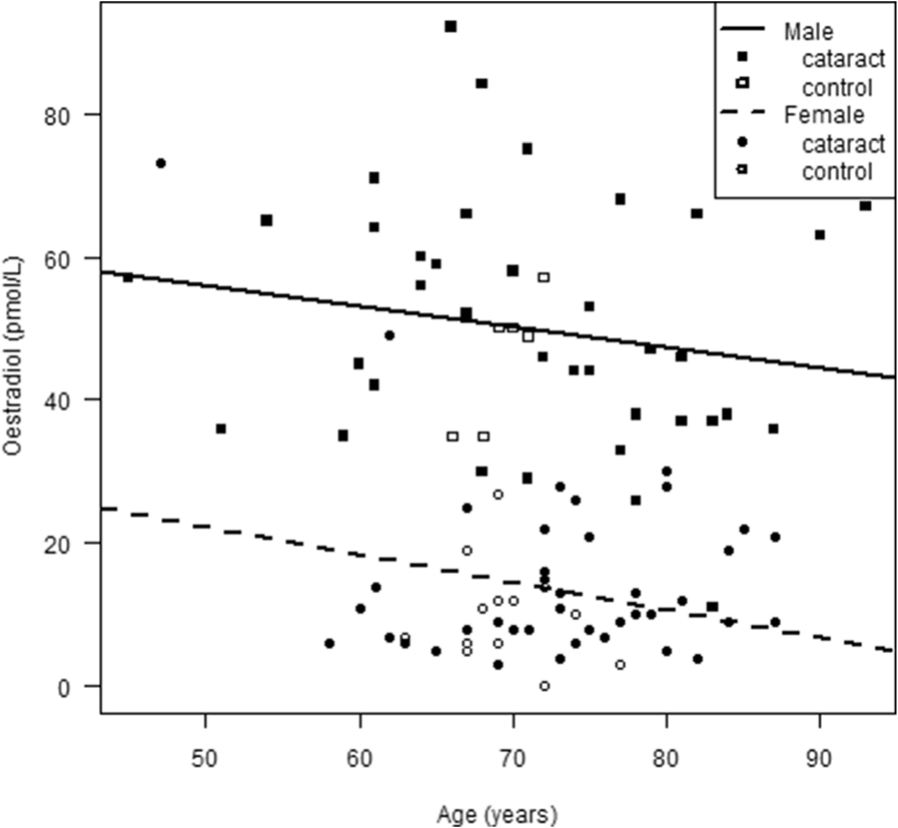
A negative correlation between age and serum oestradiol (E2) levels was seen in cataract patients and controls with linear regression analysis. Higher levels of serum E2 levels were evident in men compared to postmenopausal women

**Fig. 2:**
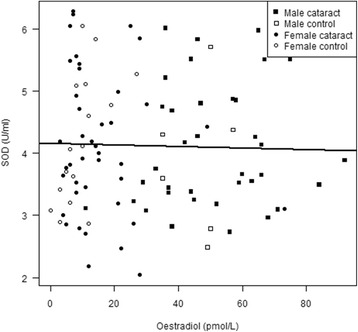
No correlation between serum oestradiol (E2) levels and superoxide dismutase (SOD) activity levels in erythrocytes was seen in the linear regression analysis of cataract patients and controls. No difference in SOD activity in erythrocytes was seen between men and postmenopausal women

There were no differences between the cataract and control group in demographic data except for a significantly higher frequency of diabetes in cataract patients compared to the control group (Table 1). Men had significantly higher E2 levels compared to postmenopausal women (*p* < 0.001); the mean level for men in both the cataract and control group (*i.e.* all subjects) was 50.1 ± 16.3 pmol/L compared to 13.8 ± 11.8 pmol/L for postmenopausal women, thus a considerable difference. E2 levels and SOD activity for the cataract and control group are shown in Table 2, with men and women presented separately. A negative correlation between age and E2 levels was seen in a linear regression analysis (Fig. 1). The higher E2 levels in men compared to postmenopausal women are also evident from the figure. Multivariate analysis showed significant impact of both age (*p* = 0.021) and sex (*p* = <0.001) on E2 levels, but not with cataract diagnosis. No correlation between SOD activity and E2 levels was found, using a multiple linear regression analysis with age, gender and diagnosis as covariates and the SOD-plate as a fixed factor in the model (Fig. 2). No predictors showed significant impact on SOD activity.

**Table 2:** Concentrations of oestradiol (E2) in serum and superoxide dismutase (SOD) activity in erythrocytes in patients with age-related cataract and controls

	Cataract		Controls	
	Men	Women	Men	Women
	*n = 37*	*n = 42*	*n = 6*	*n = 16*
Oestradiol levels (pmol/L) ^a^	50.7 ± 17.2	15.4 ± 13.0	46.0 ± 9.0	9.6 ± 6.6
	*n = 41*	*n = 60*	*n = 6*	*n = 16*
SOD activity (U/ml)	4.1 ± 0.9	4.1 ± 1.0	3.9 ± 1.2	4.2 ± 1.0

Data presented as mean ± SD

^a^ Oestradiol (17β-oestradiol) levels measured in serum are only presented for postmenopausal women

## Discussion

The main sources of circulating oestrogen are the ovaries and testes in premenopausal non-pregnant women and men, respectively. Extragonadal production, like in adipose tissue, is also of physiologic importance as source of oestrogen in both men and postmenopausal women. In the ovaries and testes, oestrogens are biosynthesized from cholesterol and testosterone respectively whereas in extragonadal sites oestrogens are derived from androgenic precursors such as dehydroepiandrosterone (DHEA), androstenediol and androstenedione, which can be converted into testosterone. Oestradiol (E2) is then biosynthesized through aromatization of testosterone and subsequently bound to sex hormone-binding globulin (SHBG) or albumin. Only a small fraction of E2 circulates free and unbound in the blood. E2 is the predominant oestrogen during the reproductive years in women and a dramatic decrease in E2 levels is seen in women entering menopause [12].

We observed significantly higher E2 levels in men compared to postmenopausal women. It has previously been reported that serum E2 levels can be higher in older men than in older women due to higher levels of testosterone and DHEA in men, resulting in higher E2 through peripheral aromatization [13, 14]. The reduction of E2 in women in menopause is thus not only dramatic, it even drops below the E2 concentration in men, something that may explain the increased prevalence of cataract in women. This hypothesis is further supported by studies on HRT and risk of cataract in women, showing protective effects of exogenously added oestradiol in postmenopausal women [4–6]. In addition, a large epidemiological study on prostate-cancer patients demonstrated higher incidence of cataract in those treated with androgen deprivation therapy, which lowers testosterone levels and consequently also decreases the production of E2 [15]. Oestradiol has been identified in the aqueous humour of humans, but the investigators did not find any differences between cataract patients and controls nor between men and women, when measuring E2 levels in aqueous humour [16]. Regarding the potential protective effects of oestradiol on cataract formation, a possible upregulation of SOD has been suggested in in vitro experiments and in animal models [7, 8]. The present study is the first to actually investigate a possible correlation between oestradiol concentration and SOD activity in humans. However, in this study no such correlation could be found in cataract patients or in controls. Alternative explanations for the protective role of oestradiol may be scavenging of reactive oxygen spices or upregulation of other antioxidant systems.

There have been reports of increased SOD activity in erythrocytes in cataract patients compared to controls and the POLA study group showed an increased incidence of cortical cataract in patients with high SOD activity in erythrocytes [17–19]. Rajkumar et al. demonstrated increased SOD activity in lens capsule samples from cortical cataract patients and the activity declined gradually with age in all samples; the highest levels of SOD were found in samples from patients 50 years of age or younger [20]. However, conflicting data on SOD activity levels also exist and there are studies showing decreased SOD activity levels in erythrocytes, sera and lenses from cataract patients compared to controls [21–23]. The study size and size of the control group are limitations in our study and no difference in SOD activity was seen between cataract and controls, but few subjects in the control group renders this comparison difficult.

## Conclusion

In conclusion, these findings are in accordance with previous reports showing higher E2 concentration in men than in postmenopausal women. Although the present data does not provide hard proofs that oestradiol protects against cataract, the results are still supportive for the hypothesis that it is the decline in oestrogen for women in menopause that may be cataractogenic. However, the results do not support that a possible beneficial effect of oestrogen acts through an E2-mediated upregulation of SOD.

## Abbreviations

DHEA: Dehydroepiandrosterone
E2: Oestradiol
EDTA: Ethylenediaminetetraacetic acid
HRT: Hormonal replacement therapy
RIA: Radioimmunoassay
SHBG: Sex hormone-binding globulin
SOD: Superoxide dismutase
